# CFD Simulations of Ventilation and Interunit Dispersion in Dormitory Complex: A Case Study of Epidemic Outbreak in Shanghai

**DOI:** 10.3390/ijerph20054603

**Published:** 2023-03-05

**Authors:** Yuwei Dai, Dongmei Xu, Haidong Wang, Fuyao Zhang

**Affiliations:** School of Environment and Architecture, University of Shanghai for Science and Technology, 516 Jungong Rd., Shanghai 200093, China

**Keywords:** COVID-19, ventilation performance, CFD, dormitory complex, infectious risk assessment

## Abstract

Since the beginning of March 2022, a new round of COVID-19 outbreaks in Shanghai has led to a sharp increase in the number of infected people. It is important to identify possible pollutant transmission routes and predict potential infection risks for infectious diseases. Therefore, this study investigated the cross-diffusion of pollutants caused by natural ventilation, including external windows and indoor ventilation windows, under three wind directions in a densely populated building environment with the CFD method. In this study, CFD building models were developed based on an actual dormitory complex and surrounding buildings under realistic wind conditions to reproduce the airflow fields and transmission paths of pollutants. This paper adopted the Wells–Riley model to assess the risk of cross-infection. The biggest risk of infection was when a source room was located on the windward side, and the risk of infection in other rooms on the same side as the source room was large in the windward direction. When pollutants were released from room 8, north wind resulted in the highest concentration of pollutants in room 28, reaching 37.8%. This paper summarizes the transmission risks related to the indoor and outdoor environments of compact buildings.

## 1. Introduction

In recent years, a global pandemic caused by COVID-19 associated with SARS-CoV-2 has occurred. This virus is believed to be transmitted mainly by close and indirect contact, as well as by airborne transmission [1]. Its mode of transmission among people has received extensive attention [2,3,4,5]. Studies have shown that small viruses encased in respiratory droplets and particles can be suspended in the air for a long distance and a long time, resulting in a significant increase in the risk of air transmission of SARS-CoV-2 in special scenarios, such as enclosed spaces, indoor environments with insufficient air treatment, etc. [6]. Except for direct contact and short-distance transmission, long-distance transmission (cross-household and cross-floor transmission) also exists. There is a special long-distance pollutant transport mode, namely interunit dispersion, which has been popularized [7]. A similar incident occurred during the SARS outbreak in Hong Kong in 2003. In less than 20 days, the number of patients in Amoy Garden reached 321. Many scholars have given explanations for this public health emergency, which was characterized by rapid transmission and a large number of infected people. The results have shown that the SARS virus is most likely to be transmitted to each household room through the air and cause residents to become infected [8]. Many subsequent studies also show that pollutants can indeed be spread by opening windows and ventilation windows.

In building dwelling units containing pathogenic aerosols, windows flush with the façade have been identified as the main route of transmission to outside [9]. This external transmission path is caused by single-sided, natural ventilation and has been comprehensively explored in terms of infectious risk through field measurements, numerical simulations, and wind tunnel experiments. Niu and Tung [10] found that exhaust from the bottom 7% could re-enter upper rooms due to single-sided, natural ventilation through buoyancy effects, suggesting that aerosols carrying pathogens could be transmitted from windows on the same side of a lower floor to upper floors. The effects of outdoor window flush and pathogen transmission were also analyzed [11]. When the wind effect is dominant, window flush pathogens can spread vertically and horizontally [12]. In 2003, the SARS virus broke out in Hong Kong, and the number of people infected in Amoy Garden increased dramatically in a short time. Yu et al. [8] used CFD and a multiregion model to analyze whether there was a close relationship between the distribution of infected people, the airflow direction between floors, and the wind velocity field in the community, which indirectly proved the role of airborne transmission. Ai et al. [12,13,14] carried out a series of numerical simulations to quantify the exhaust re-entry ratio from each different room to other rooms in a multistory building and revealed the possible transmission path of pollutants. Dai et al. [15] investigated the effect of changes in airflow patterns on interunit dispersion characteristics around multistory buildings due to wind effects and quantitatively assessed the potential risk of different routes for interunit dispersion. Li et al. [16] used a multiregion airflow-modeling method to analyze the dispersion of aerosols between flats in the area of leakage through the doors and windows of a typical building under six different scenarios, identifying possible environmental causes of airborne transmission associated with the airflow between flats. Wu et al. [17] studied the spread of respiratory infectious diseases and assessed the risk of cross-infection in a typical high-rise residential environment with the CFD method, focusing on the spread between buildings under the effect of wind. It was concluded that the risk of cross-infection between buildings could not be ignored, especially when there were highly infected people with high pathogen production rates. Recently, Dai et al. [18,19] conducted a series of outdoor scale model experiments to study the problem of pollutant transmission between households in street canyons under real urban atmospheric conditions. These studies have shown that pollutants are likely to spread between rooms in the same building and between different buildings. However, a dormitory complex with corridors at a typical university campus has the characteristics of compact space, short distance, and close connections. In such a high-risk environment, studies of pollutant transmission are not comprehensive. Therefore, according to the outbreak of the epidemic in March in Shanghai and the local weather conditions, this paper simulates virus transmission in a dormitory complex at a typical location using the CFD method and evaluates the infectious risk.

In addition to transmission through external windows, pollutants can also diffuse internally between horizontally adjacent rooms on the same floor. Transmissions between rooms and corridors or between rooms sharing the same corridor have been reported in isolated hotels. A similar case was reported in Hong Kong in a quarantine hotel, where one guest caught the SARS virus from two people in the room opposite the corridor without direct contact because of insufficient ventilation [20]. Jiang et al. [21] demonstrated that extensive environmental contamination with SARS-CoV-2 RNA was detected in a relatively short time (<24 h) in the occupied rooms of two people who were presymptomatic. Therefore, it is important to study transmission through the interior of a building. Wu et al. [22] studied the influence of airflow in buildings due to the specific configuration of semi-open corridors using tracer gas. The study showed that the wind effect and semi-open corridor conditions played a major role in transmission. They concluded that the airflow in the semi-open corridor promoted the rapid dilution of pollutants, prevented the accumulation of pollutants, and effectively reduced the dispersion between devices. Cheng et al. [23] estimated the airflow rate and aerosol concentration in an apartment and corridor on the same floor as the pollution source through the CFD method. It was concluded that the apartment opposite the corridor and adjacent to the apartment downstream of the pollution source had the highest exposure risk under the prevailing east wind, especially when the doors and windows connected to the corridor were open. The results provided a possible explanation for outbreaks of COVID-19 in isolated hotels. Positive pressure and adequate ventilation in corridors can help reduce such cross-corridor infections. Wu et al. [24] studied the internal diffusion path between adjacent horizontal planes caused by air infiltration through field measurement, closed window mode, and opened window mode and found that the concentration of tracer gas in the receptor room was one order of magnitude lower than that in the source room, and the risk of infection was also one level lower. The relative risk of cross-infection through internal transmission could be 9%, which was higher than external transmission through a single window. This implied that horizontal transmission routes caused by air infiltration should not be underestimated. Through experiments and CFD simulations, Zhang et al. [25] put forward a method of providing cross-ventilation for classrooms on both sides by setting horizontal airflow channels in buildings, which is used to improve the natural airflow rates of other buildings with indoor corridors. These contributions to the literature have studied the spread of pollutants inside buildings but not in an actual situation where the windows on either side of a corridor on a dormitory floor are open. This paper investigates the concentration levels of viruses in other rooms on the same floor when different dormitory rooms are infected, with dormitory doors closed and ventilation windows and external windows (external windows of rooms and windows on both sides of the corridor) open.

Field measurements, wind tunnel experiments, and CFD methods are commonly used to study ventilation and pollutant dispersion. Full-scale field measurement takes into account all airflow and diffusion field phenomena under real atmospheric conditions. However, field measurements have certain limitations and difficulties, such as limited locations to collect data and uncontrollable wind and weather conditions. Unlike field measurements, wind tunnel experiments allow a high degree of control over boundary conditions. However, due to the limitations of physical boundaries, reduced scale models are usually used for wind tunnel experiments, which may lead to similarity problems [26,27]. Compared to field tests and wind tunnel experiments, CFD simulations have become a widely used research method by researchers due to their ability to fully control boundary conditions and provide unrestricted data on the entire flow field [28]. At the same time, these simulations are not limited by weather conditions and similarity requirements. In addition, many CFD studies use a scaled-down model to study flow structure and pollutant dispersion in urban environments to save computational resources. Compared with large eddy simulation and detached eddy simulation models, the modeling method based on RANS has advantages in computing resource consumption [29,30]. Plenty of studies have successfully used RANS models to predict indoor and outdoor airflow under leeward and windward conditions [31,32]. It has been proved that a scaled-down CFD model and a steady RANS model can make accurate predictions and save a lot of money and calculation time in computing costs [33,34,35,36,37,38]. Robert et al. [39] compared simulation and wind tunnel experimental results using standard k-ε and RNG k-ε and showed that RNG k-ε had a higher accuracy for flow and concentration field prediction compared to standard k-ε. Therefore, this study is conducted based on the most widely used RNG k-ε turbulence model to simulate a COVID-19 outbreak in a dormitory complex in Shanghai.

In order to further study interunit dispersion, this paper takes a dormitory complex in Shanghai as a prototype, using the CFD method to simulate the dispersion of pollutants in the rooms. N_2_O is used as a tracer gas to simulate the dispersion of the virus driven by different wind directions (north wind, southeast wind, and northwest wind) in three environments (as is shown in Section 4: Dormitory 3, quarantine area 1, and inside). The pollutant dispersion problems between coupled indoor and outdoor airflow driven by single-sided, natural ventilation and pollutant dispersion inside and around naturally ventilated buildings are analyzed. In this paper, an RNG k-ε turbulence model is used to simulate the propagation path of the virus in a dormitory complex, and the transmission mechanism of pollutants is analyzed according to the simulation results, which provides theoretical support for future virus transmission research and epidemic prevention and control measures. The innovation of this research includes two points. First, the cross-diffusion paths of pollutants caused by natural ventilation (including external and internal ventilation windows) are investigated in a compact living environment (school dormitory), which is a typical high-risk condition. Then, this paper considers three realistic wind directions based on an actual dormitory complex and surrounding buildings. Section 2 describes the CFD model and solution method. Section 3 validates the CFD model. Section 4 introduces the configuration description. Detailed results and discussions are presented in Section 5. Section 6 summarizes and concludes the study.

## 2. Methodology

### 2.1. CFD Model

The CFD approach is commonly used to predict airflow patterns and pollutant dispersions. The governing equations in RANS models for incompressible Newtonian fluids can be written as follows:

(1) $$\frac{\partial \bar{u_{i}}}{\partial x_{i}}=0$$

(2) $$\frac{\partial \left(\rho \bar{u_{i}}\right)}{\partial t}+\frac{\partial \left(\rho \bar{u_{i}u_{j}}\right)}{\partial x_{j}}=-\frac{\partial \bar{p}}{\partial x_{i}}+\vartheta \frac{\partial^{2}\bar{u_{i}}}{\partial x_{i}^{2}}-\frac{\partial \tau_{ij}}{\partial x_{j}}$$

(3) $$\frac{\partial \left(\rho \bar{c}\right)}{\partial t}+\frac{\partial \left(\rho \bar{u_{j}c}\right)}{\partial x_{j}}=-\frac{\partial J_{j}}{\partial x_{j}}$$

where $u_{i}$ and $u_{j}$ represent velocity components, ρ is the density, ϑ represents the viscosity,  p represents the pressure, $\tau_{ij}$ is the subgrid-scale (SGS) stress, and the overbar (-) indicates time-averaged components.

In this study, an RNG k-ε model is used with improvements on the standard k-ε model [40,41,42,43]. The term$\;R_{\varepsilon }$, as an additional strain dependent on the transport equation, makes the RNG model more sensitive in dealing with fast strains and streamlined curvature compared to the standard k-ε model.

The added term $R_{\varepsilon }$ is shown by the equation as follows:

(4) $$R_{\varepsilon }=\frac{C_{u}\rho \eta^{3}\left(1-\eta /\eta_{0}\right)}{1+\xi \eta^{3}}\cdot \frac{\varepsilon^{2}}{k}$$

where $C_{u}$, $\eta_{0},$ and ξ are the constants of the model, and η≡Sk/ε, where *S* is referred to as the strain rate scale. The discretization of the control equations is key to the numerical simulation calculations and is usually discretized into algebraic equations on a staggered grid, followed by a coupled solution.

### 2.2. Solution Method

ANSYS 2020 R2 was adapted for the simulation research on buildings. The finite volume method was used as the numerical solution method for this simulation. The SIMPLEC algorithm was used for pressure–velocity coupling. For the convection, diffusion term, and pressure difference method, the second-order precision discrete scheme was adopted. The simulation was considered to achieve the purpose of convergence when all scalar residuals were less than 10^−5^, and computational stability was achieved in several iterations.

## 3. Validation of CFD Method

### 3.1. Validation of Airflow Field

**Figure 1:**
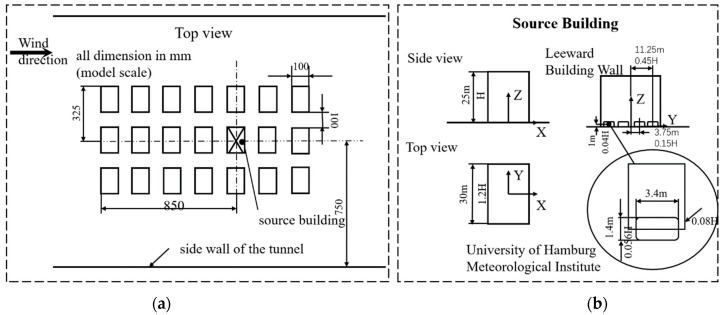
(**a**) Dimensions of building arrays, (**b**) source building and source emissions [45].

In order to validate the physical model of the airflow field, experimental measurements of the airflow and diffusion field of a 3 × 7 rectangular building array model (CEDVAL B1-1) were carried out in a Blasius wind tunnel at the University of Hamburg. The geometry is depicted in Figure 1a.

Four sources were set up on the leeward side of the target buildings, and the pollutants were emitted at a velocity of 0.025 m/s, as shown in Figure 1b. The laser doppler velocimetry (LVD) technique and a flame ionization detector (FID) were used to measure the airflow and concentration field, respectively. In this study, the computational domain shown in Figure 1 was used to simulate the flow field and diffusion field around the building model. The simulation results were compared with wind tunnel test data to demonstrate the feasibility of the CFD method. The size of the computational domain selected was based on existing best practice guidelines [44]. The distance between the left boundary (entrance) and the first building was 5 H, the distance between the right boundary (exit) and the last building was 15 H, and the height from the top of the highest building to the upper boundary was 5 H. According to the width of the wind tunnel, the lateral distance of the calculation area was 3.4 H. Using the boundary conditions in Table 1 to generate the inlet flow, the coefficients were $z_{0}=0.00075\;m$, $u^{*}=0.4078\;m/s$, $C_{1}=0.41$, κ=0.4187, and $C_{u}=0.069$.

A grid sensitivity analysis was carried out to select a suitable computational grid to generate grid-independent results for the CFD simulation. Three mesh systems were constructed using coarse, medium, and fine unstructured tetrahedral meshes. The numbers of grids were about 3.2 million, 5.9 million, and 9.3 million, respectively. The independence of the grids was established with a grid convergence index (GCI), as expressed by Equation (5) and proposed by Roach [46]:

(5) $$\mathrm{GCI}=F_{s}\left|\frac{r^{p}\left(u_{i,grid_{A}}-u_{i,grid_{B}}\right)/U_{ref}}{1-r^{p}}\right|$$

where $F_{s}$ is the safety factor, which is 1.25, because three computational grids were used in the grid sensitivity analysis. *r* is the mesh refinement degree and is equal to $\sqrt{2}$. The medium mesh was more refined than the coarse mesh and, in turn, was thicker than the fine mesh, with the same coefficient. *p* is the order of formal accuracy, which was taken as 2 because all the CFD simulations used the second-order discrete scheme. $u_{i}$ represents the *U* wind speed measured in the grids, and $U_{ref}$ is the reference wind speed, which is equivalent to 5.67 m/s.

The GCI for each location was computed by pairing the medium and coarse grids’ $U/U_{ref}$ values, as well as pairing the medium and fine grids’ values. The variation in GCI with height varied in three locations, and the average heights of the medium-fine grid were 0.77%, 0.86%, and 1.90% in three locations. For the medium-coarse grid, the values were 1.55%, 3.51%, and 2.18%. Since the average height GCI values of these three grids were less than 5%, any of these grids could be used for the CFD simulation.

**Figure 2:**
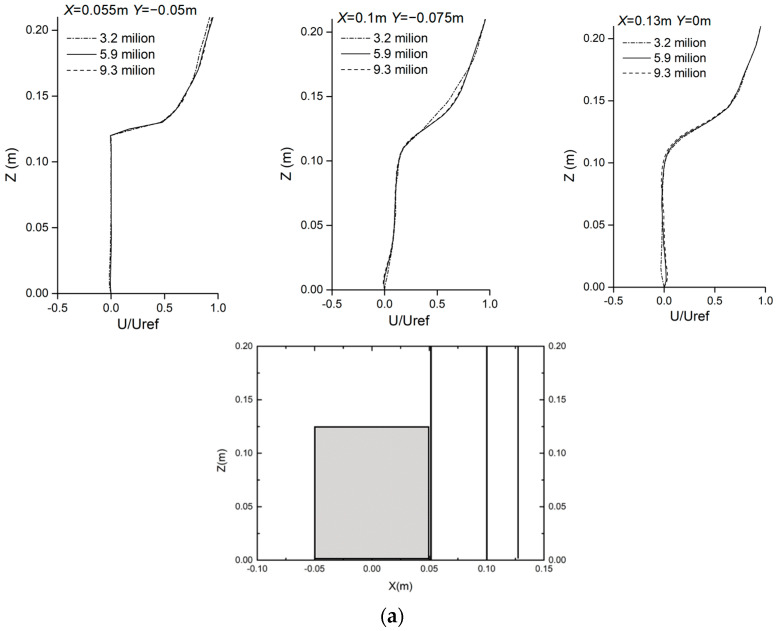

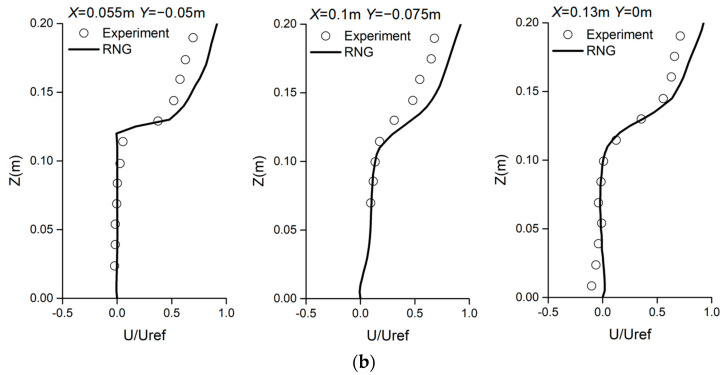
Comparison between grid−independent solution verification and experiment data. (**a**) Comparison of mean velocity profiles using three types of mesh systems at the vertical line. (**b**) Comparison of mean velocity profiles using medium grid and experiment data at the vertical line.

Figure 2a shows the results of the grid sensitivity test, which was constructed based on dimensionless wind speeds on vertical lines at three different locations in front of the target building. The results show that the system with 3.2 million grids showed a considerable underestimation compared to the results of the models with 5.9 million and 9.3 million grids, while the results for 5.9 and 9.3 million grids were essentially equivalent. Therefore, the medium mesh system with 5.9 million grids was selected for its efficiency in using computational resources and high accuracy.

Figure 2b is a comparison of mean velocity profiles using medium grids and experiment data at the vertical line. As shown in Figure 2b, the CFD results agreed well with the experimental data regarding wind flow velocity. Therefore, this paper used this simulation method to simulate the ventilation and pollutant diffusion of buildings.

## 4. Configuration Descriptions

**Figure 3:**
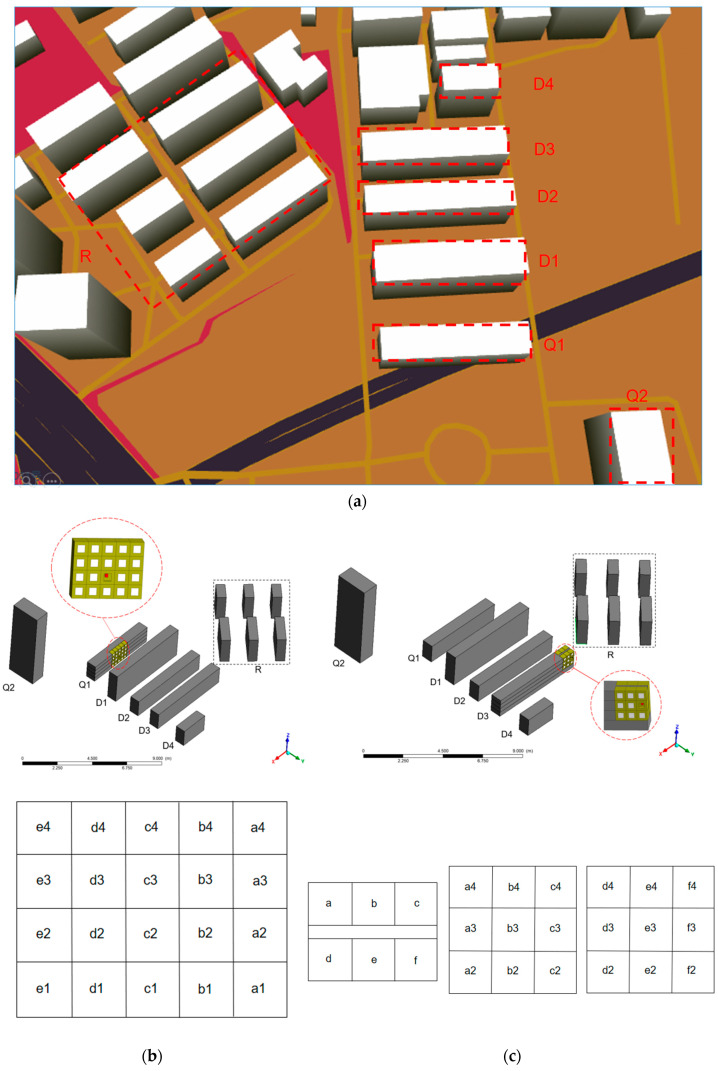

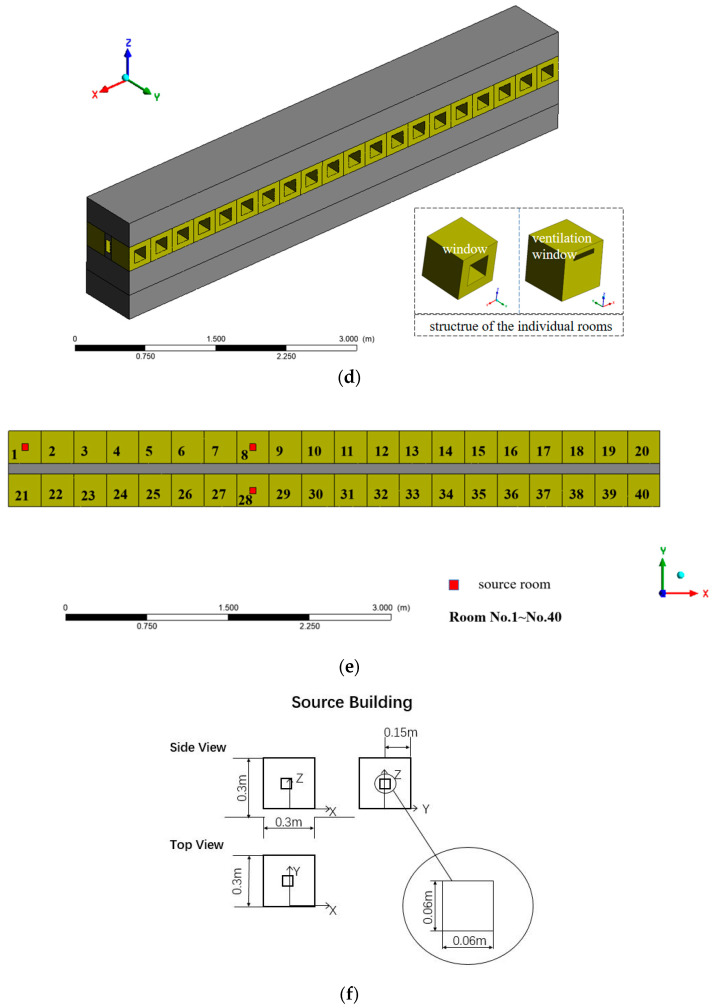
Building arrangements of dormitory complex. D: Dormitory building; Q: Quarantine area; R: Residential building. (**a**) Building layout near the dormitory complex location. (**b**) Schematic diagram of Q1. (**c**) Schematic diagram of D3. (**d**) Physical model of the third floor of D3. (**e**) Plan view of the third floor of D3. (**f**) Setting of the pollution source in the room.

In order to investigate the ventilation and pollutant dispersion in a coupled indoor and outdoor environment, three 1:10 reduced-scale building models were constructed. This study simulated a dormitory area, a quarantine area, and a surrounding environment. In the present study, the simulated model included 12 buildings with one pollutant source located in the target building, the physical configuration of which is shown in Figure 3. In this figure, D represents the dormitory building, Q represents the quarantine area, and R represents residential buildings. There were four dormitory buildings (D1, D2, D3, and D4) for students to live in. If a student in a dormitory building was infected, the individual was transferred to the quarantine area. The six buildings near the dormitory complex were residential buildings outside the campus that affected the surrounding wind field. The dimensions of the D3 model were length × width × height = 6 × 0.7 × 1.2 m. The dimensions of the Q1 model were length × width × height = 5.1 × 0.7 × 1.2 m. This study chose buildings D3 and Q1 as target areas, respectively. The window dimensions of the target buildings were length × height = 0.16 × 0.16 m. The ventilation window dimensions of the target buildings were length × height = 0.15 × 0.07 m.

The virus was not only transmitted through the dormitory windows but also spread between the corridors through the ventilation windows between floors. To solve the problem of pollutant dispersion inside building D3, the third floor of D3 was modeled, as shown in Figure 3d. There were 40 rooms on this floor, and each room had an external window for natural ventilation. At the same time, there was a ventilation window above the dormitory door. Open windows were also set on both the left and right sides of the corridor for ventilation. Room 1, Room 8, and Room 28 were used as rooms for infected patients to explore the path of virus spread on the floor.

**Figure 4:**
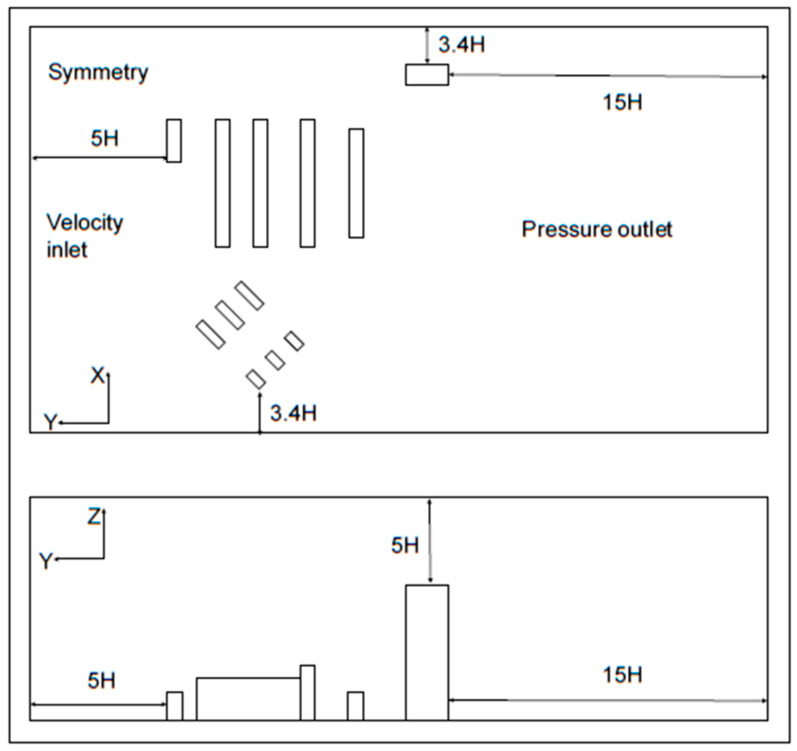
The arrangement and computational domain of twelve buildings.

**Figure 5:**
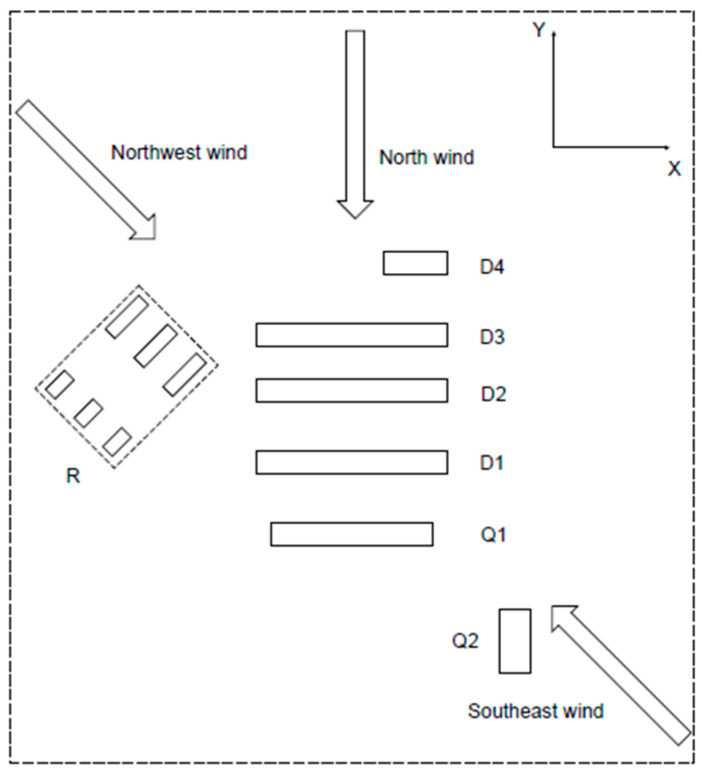
Schematic diagram of dormitory complex under three wind directions. D represents the dormitory building, Q represents the quarantine area, and R represents residential buildings.

The computational domains of three cases were constructed following the validation in Section 3.1, and the domain of the dormitory complex is shown in Figure 4. The wind direction was perpendicular to the inlet plane and the opening of the room. In this study, three wind directions were selected based on meteorological data of Shanghai from March to April, as shown in Figure 5, including north wind, southeast wind, and northwest wind, and the wind velocity was 3.8 m/s at 10 m. The transmissions of pollutants driven by the three wind directions in the three environments were simulated, respectively. Taking one of the three layout forms as an example, as shown in Figure 5. The value of H is the average value of the height of twelve buildings, equal to 1.925 m, the distance between the inlet and the first building was 5 H, the distance between the outlet and the last building was 15 H, and the height from the top of the highest building to the upper boundary was 5 H. The boundary conditions were adopted from Table 1.

As shown in Figure 3f, the source model was placed in the middle of the building. When the pollutant dispersion was simulated, the tracer gas was continuously released from the source element. N_2_O tracer gas [47] was selected, and the source intensity was set as 0.013 $\mathrm{kg}/m^{3}\cdot s$ to simulate the virus dispersion range. The source intensity was set based on the average exhalation rate of the human body, which is 12 L/min [48], as well as the volume fraction of aerosol with virus in human exhaled airflow, which is about 4% [49]. No perturbations were considered for the emission of pollutants.

**Figure 6:**
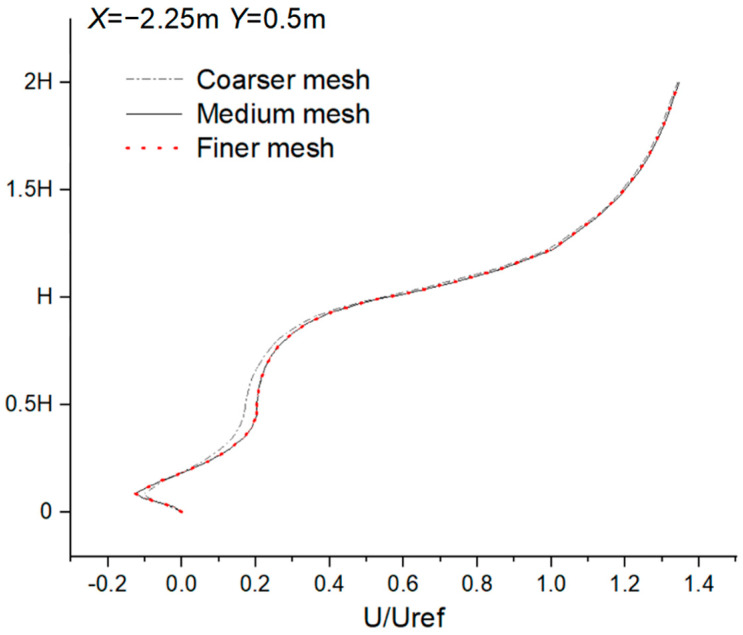
Comparison of mean velocity profiles using three types of mesh systems at the vertical line of X = −2.25 m, Y = 0.5 m.

A grid-independent validation was carried out in order to simulate the ventilation and pollutant dispersion in the dormitory complex. Three meshes were compared, containing approximately 2.67, 4.0, and 5.6 million unstructured tetrahedral meshes (see Figure 6), and the medium mesh (4.0 million) was chosen, taking into account numerical accuracy and cost.

## 5. Results and Discussion

### 5.1. Dormitory Complex

#### 5.1.1. Airflow Field

**Figure 7:**
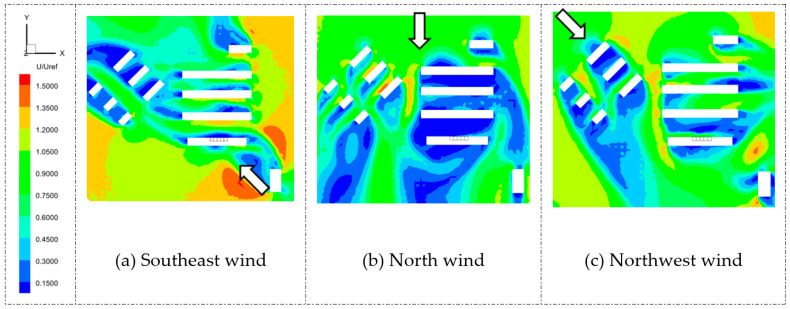
Contours of mean velocity on horizontal planes of Q1 at the height of 0.46 m under three wind directions. Arrows represent three different wind directions.

**Figure 8:**
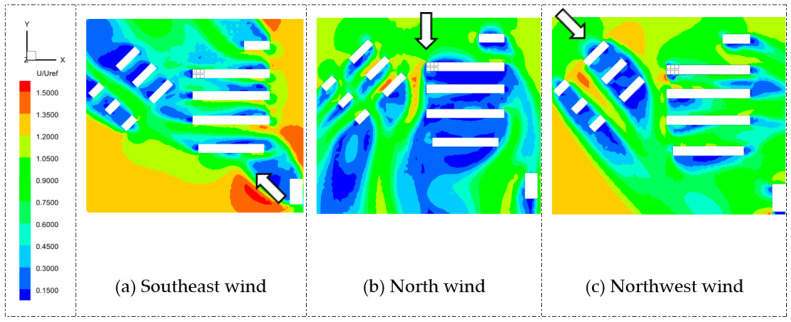
Contours of mean velocity on horizontal planes of D3 at the height of 0.76 m under three wind directions. Arrows represent three different wind directions.

The airflow distribution around a building complex affects the pollutant dispersion between rooms. Figure 7 and Figure 8 show the average wind velocity contours of Q1 and D3 at the middle heights of the source rooms with three wind directions. Note that the three wind directions were selected based on meteorological data of Shanghai from March to April. It is clearly shown that, under north wind conditions, the airflow field around the dormitory complex had a large, low-speed area, while under the southeast and northwest wind conditions, the wind velocities around the target buildings were relatively high. Due to the oblique wind directions, the airflow fields were highly changed and induced a large, high-speed zone in the near-wall area, which may affect the pollutant distribution around the building complex.

In addition, for Q1, the most unfavorable wind condition was the north wind direction. When the wind was blowing north, the upstream buildings of the Q1 target building were six residential buildings and four dormitory buildings, as referred to in Figure 5. The wind velocities on both the windward and leeward sides of Q1 were low, which implied that the pollutant released from Q1 may not disperse quickly, resulting in higher interunit transmission.

#### 5.1.2. Concentration Field

**Figure 9:**
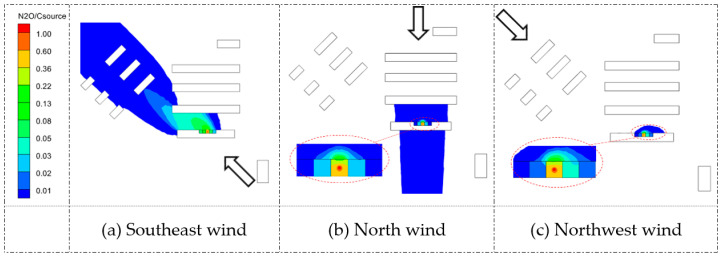
Comparisons of nondimensional concentration fields on horizontal plane Z = 0.46 m under three wind directions for Q1 source building. Arrows represent three different wind directions.

**Figure 10:**
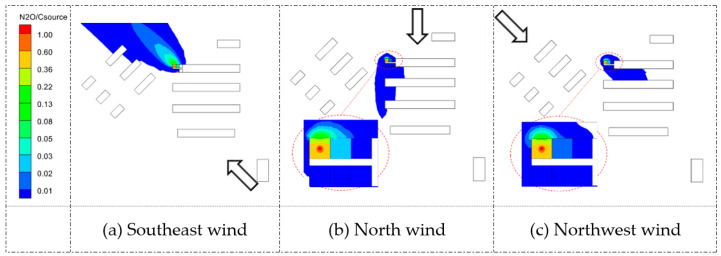
Comparisons of nondimensional concentration fields on horizontal plane Z = 0.76 m under three wind directions for D3 source building. Arrows represent three different wind directions.

Figure 9 and Figure 10 display the nondimensional concentration contours of pollutant dispersion around the building complex, which was calculated with $\frac{C_{N_{2}O}}{C_{source}}$, where $C_{N_{2}O}$ is the tracer gas concentration of the dormitory complex, and $C_{source}$ is the tracer gas concentration at the source. A non-dimensional concentration level lower than 0.01 was considered negligible.

Generally, in such dormitory complexes, pollutants released from source buildings barely affect surrounding buildings. The largest affected condition was the source room located in Q1 with the southeast wind, as shown in Figure 9a, with the windward side of D1 (opposite the source room) suffering slight pollutants. In addition, larger affected areas implied that the pollutants dispersed more easily, and the ventilation of the target building was more effective under this situation. Overall, the pollutant transmission to other buildings could be considered negligible.

However, pollutant dispersion within a source building cannot be neglected. As shown in Figure 9 and Figure 10, the concentration fields in the nearby rooms were amplified. The re-entry concentration of a certain room could be one order higher than other rooms, as shown in Figure 9b,c. These results were consistent with the airflow field in Figure 7b. The low-wind area near the building façade further implied that pollutants released from Q1 may re-enter the adjacent rooms.

#### 5.1.3. ACH and Re-Entry Ratio

##### Ventilation Rate

Air change rate (*ACH*) is widely used to evaluate room ventilation performance. It is defined as the ratio of room ventilation volume to the room volume in unit time. The *ACH* ($h^{-1}$) value calculated by the area division method is as follows [50,51]:

(6) $$ACH=3600\times \frac{0.5\int_{0}^{S_{W}}\left|V_{x}\right|dS_{W}}{Vol_{R}}$$

where $S_{W}$ is the surface area of room windows in $m^{2}$, $V_{x}$ is the vector velocity perpendicular to the window opening in m/s, and $Vol_{R}$ is the room volume in $m^{3}$.

**Figure 11:**
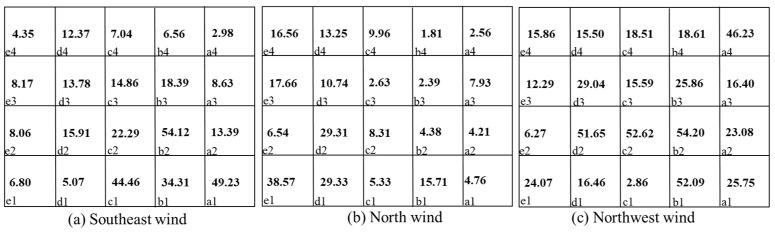
Ventilation rates of rooms *ACH* ($h^{-1}$) in Q1. a1,2,3,4-e1,2,3,4 represent the number of dormitory room.

**Figure 12:**
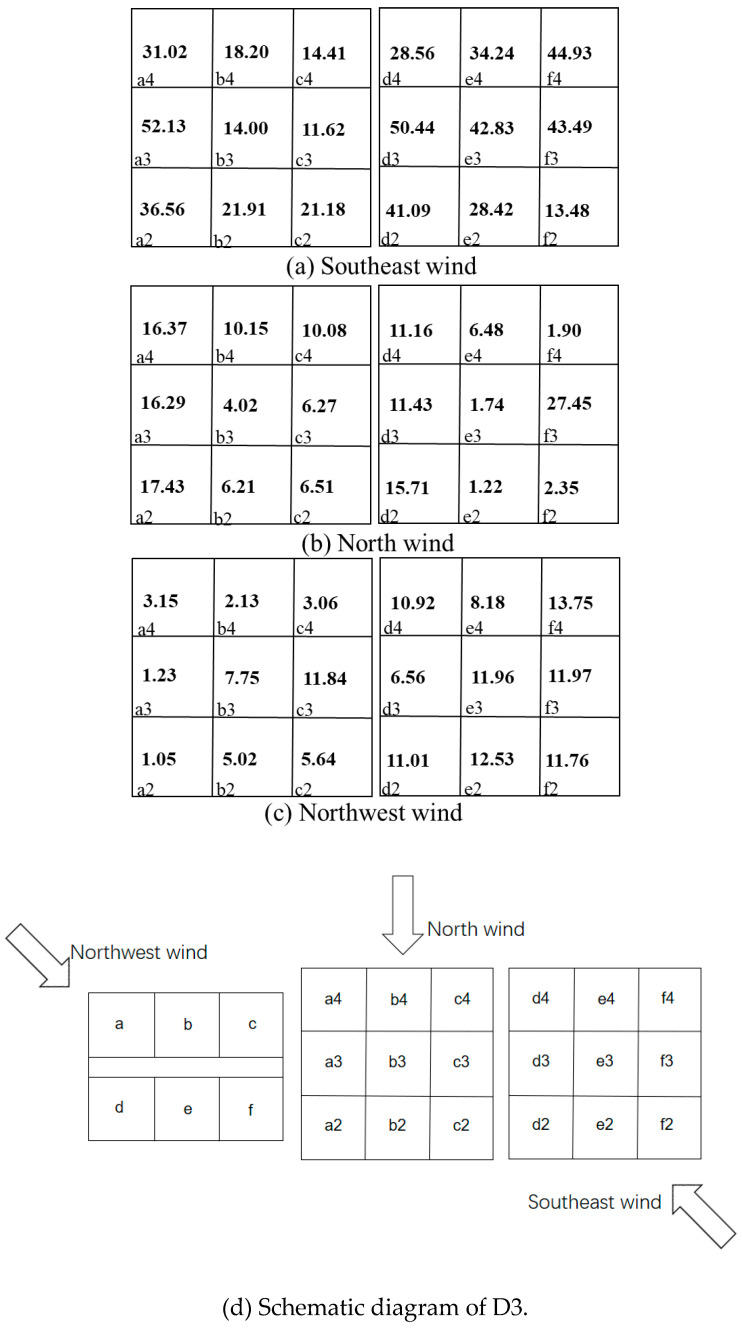
Ventilation rates of rooms *ACH* ($h^{-1}$) in D3. a1,2,3,4-e1,2,3,4 represent the number of dormitory room.

Figure 11 and Figure 12 list the *ACH* values for the individual rooms of the Q1 and D3 target buildings under the three wind directions. Different wind directions changed for buildings upstream of the target buildings, which affected the *ACH* values. When the wind direction was oblique, the *ACH* values were basically larger in most rooms of Q1 and D3 than when in the perpendicular direction. Under the northwest wind direction, the *ACH* values of most rooms in Q1 were even larger, with a maximum value of 54.2 $h^{-1}$ (room b2). From the airflow and concentration results, room position had an essential impact on the ventilation rate. With changes in the three wind directions and the upstream buildings of the target buildings, the interactions between the indoor and outdoor airflow patterns were redistributed, resulting in different airflow characteristics and pollutant concentrations.

##### Re-Entry Ratio

For naturally ventilated buildings in a dormitory complex, the pollutants released from a room re-enter other rooms in the building. In this paper, the re-entry ratio ($R_{K}$) was used to evaluate the cross-household transmission of pollutants, which is defined as the ratio of polluted air discharged from a source room i where pollutants are located to the pollutants that re-enter other rooms j, which can be calculated by the following formula [10,14]:

(7) $$R_{K}=\frac{C_{j}Vol_{j}{\left(ACH\right)}_{j}}{C_{i}Vol_{i}{\left(ACH\right)}_{i}}$$

where C is the pollutant concentration in the room in $\mathrm{kg}/m^{3}$, and i and j represent the pollution source room and the polluted room, respectively. Here, the pollutant concentration in a room is represented by that found on the respiration plane (in a standing situation) at the height of 0.16 m (1.6 m in the prototype) above the floor.

**Figure 13:**
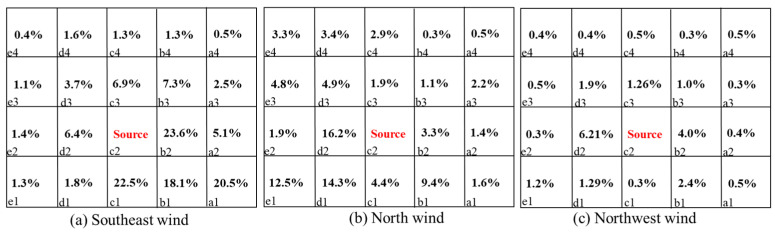
Re-entry ratio ($R_{K}$) of each room under three wind directions with the pollutant source located in room c2 in Q1.

**Figure 14:**
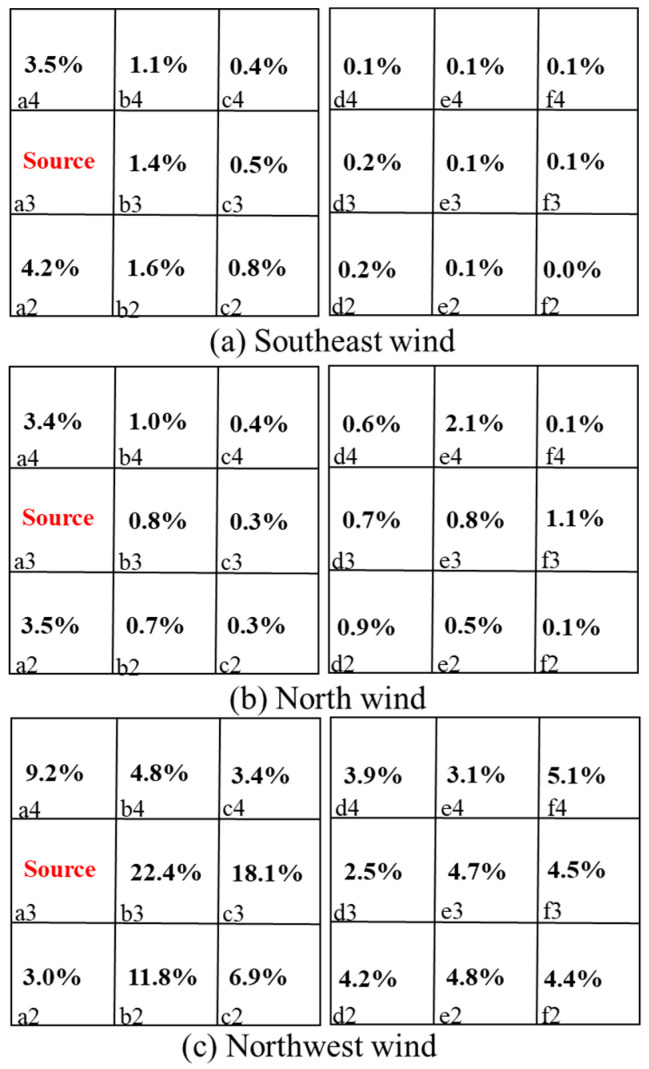
Re-entry ratio ($R_{K}$) of each room under three wind directions with the pollutant source located in room a3 in D3. a1,2,3,4-e1,2,3,4 represent the number of dormitory room.

This section discusses the patterns of pollutant dispersion in a dormitory complex when pollutants are released from two sources under three wind directions. When the wind direction changed, the airflow field near the target building varied due to the asymmetrical layout of the building complex, which led to different wind velocities and pollutant transport routes. Figure 13 and Figure 14 show the re-entry ratios of each room when the pollutants were released from certain rooms.

Several observations can be made based on the comparisons of the $R_{k}$ results. Firstly, for Q1 it can be seen from Figure 13 that when the source was located in room c2, the re-entry ratios varied greatly with wind direction. When the wind blew from the southeast direction, the re-entry ratios of all the rooms on the same side as the source room were considerably larger than for the other two directions. The highest re-entry ratio could be up to 23.6%. Under this condition, the low-wind area near the wall prevents the pollutant from dispersing quickly, as shown in Figure 7a. For the other two wind directions, the re-entry ratios decreased drastically to the highest value of 16.2%.

Secondly, for the rooms on the same side as the source room in Q1, relatively large re-entry ratios generally occurred around the source position at short distances. When the distance became further, the values of the re-entry ratio descended remarkably. The highest value occurred in the nearest room to the source position along the wind direction.

Thirdly, for D3, when the pollutant was released from a room on one side of the building, the pollutants barely dispersed to rooms on the other side. The re-entry ratios of the rooms located on the other side were lower by one or two orders of magnitude than rooms on the same side, which could be considered negligible. However, with the most unfavorable condition under the northwest wind direction, both the windward and leeward sides of the building suffered elevated re-entered pollutants. The re-entry ratios of the rooms located on the opposite side could even be up to 4.8%.

In addition, when the pollutant sources were located in different positions in such a building complex, the most unfavorable conditions could be diverse for individual buildings.

#### 5.1.4. Interunit Infectious Risk Assessment

The re-entry ratio $R_{k}$ is a parameter that describes the amount of air pollutants released from one unit to another. The risk of infection *P* describes the probability that people may be infected when exposed to a specific concentration of an air pollutant. The probability of people being infected increases as the value of *P* increases. These two scalars represent different emphases but are mathematically related.

The infectious risk between units can be evaluated by Equation (8), which is based on the Wells–Riley model [52]:

(8) $$P=1-e^{\frac{-C_{d}Iqpt}{Q}}$$

where *q* is the quantum productivity rate ($h^{-1}$) produced by an infected individual, *I* is the number of infected individuals, *t* represents the exposure time, *Q* is the ventilation rate of the rooms in each unit ($m^{3}/h$), $C_{d}$ is the concentration decay coefficient and is equal to the mass fraction of concentration ($\frac{C_{j}}{C_{i}}$), and *p* is the human pulmonary ventilation rate. When a highly infected person spreads pollutants in a target building, the transmission risk between units is very high [23]. Assuming that an infected person is present in a unit, the quantum generation rate is 48 quanta per hour [53]. When people sit indoors or participate in light activities, human pulmonary ventilation is 0.3 $m^{3}/h$ [54], *I* = 1, and the exposure time is 8 h.

**Figure 15:**
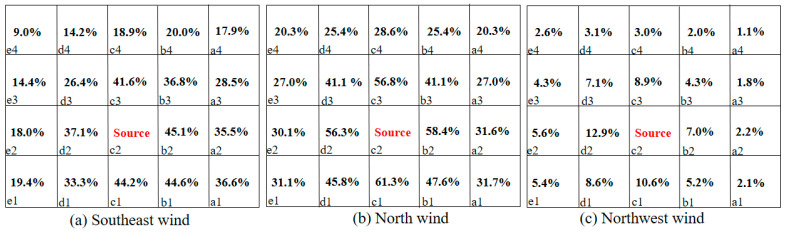
Interunit infectious risk *p* values of rooms in Q1 with room c2 as the source under three wind directions. a1,2,3,4-e1,2,3,4 represent the number of dormitory room.

**Figure 16:**
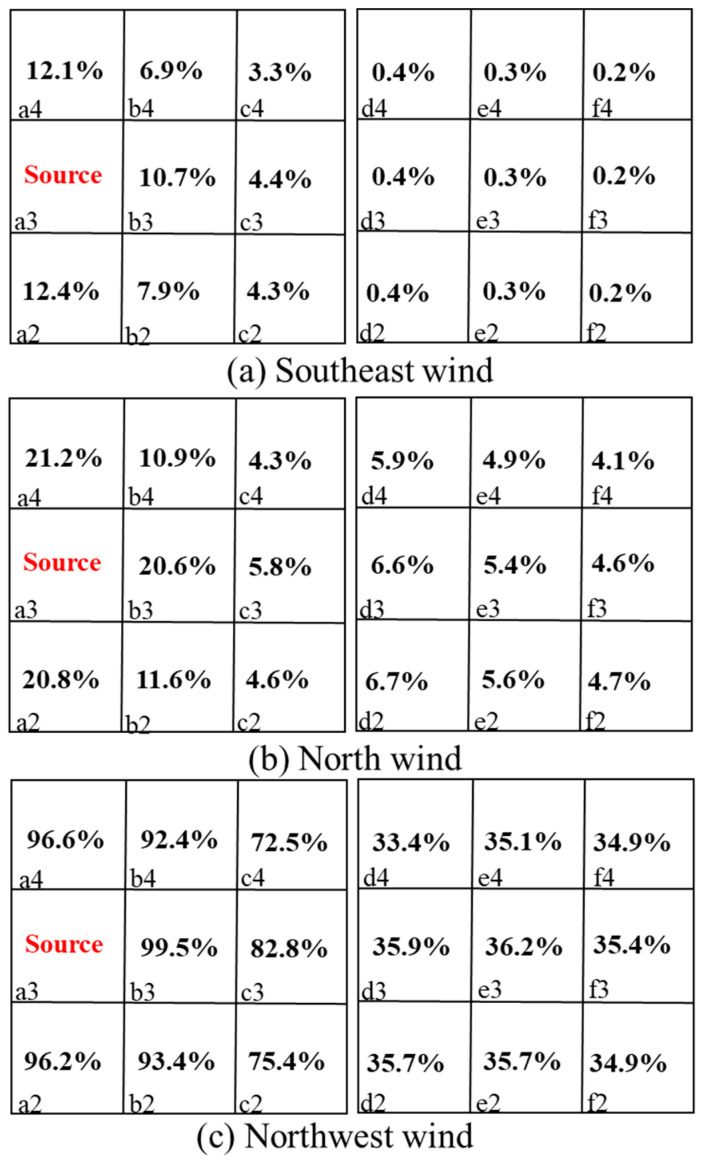
Interunit infectious risk *p* values of D3 with room a3 as the source under three wind directions. a1,2,3,4-e1,2,3,4 represent the number of dormitory room.

By introducing the *ACH*, the relation can be written as Equation (9):

(9) $$P=1-e^{\frac{-Iqpt}{V{\left(ACH\right)}_{i}}\cdot \frac{{\left(ACH\right)}_{i}}{{\left(ACH\right)}_{j}}\cdot R_{k}}$$

where ${\left(ACH\right)}_{i}$ is the air exchange rate of the source unit *I*, and ${\left(ACH\right)}_{j}$ is the re-entry unit *j*. The interunit infectious risk evaluation is listed in Figure 15 and Figure 16.

Figure 15 and Figure 16 calculate the interunit infectious probabilities for Q1 and D3 with different sources. Since the terms *I*, *q*, *p*, *t*, and *V* are constants under a certain circumstance, the term $\frac{{\left(ACH\right)}_{i}}{{\left(ACH\right)}_{j}}$ affects the relation between *P* and $R_{k}$ significantly. When a pollutant source location is fixed, the ${\left(ACH\right)}_{i}$ is a determined value, which makes ${\left(ACH\right)}_{j}$ a key factor in this scenario. If a re-entry unit has a smaller ACH under the same situation, people become infected more easily. According to the Wells–Riley model, the infectious risk of each room was relatively high when the pollution source was located in Q1 with southeast wind. Room b2 had the highest risk of transmission at 45.1%, as it was located immediately under the source room. When the pollution source was located in D3, the infectious risk of each room was relatively high with northwest wind. As the source room was on the windward side, the risk of infection in other rooms on the same side as the source room was larger than 90% with a windward direction. This may be due to a low level of ventilation in rooms other than the source room.

In other words, the natural ventilation efficiency of a re-entry unit significantly affected the infectious risk. The infectious risk increased as the ACH ratio between the source and re-entry rooms decreased. Therefore, both the re-entry ratio and infectious risk should be considered when confronting the problem of diffusion between units.

### 5.2. Inside Building D3

#### 5.2.1. Airflow Field

In addition to spreading through external windows, windows on both sides of the corridor in a dormitory building and ventilation windows above interior doors can also cause pollutants to spread internally between horizontally adjacent rooms sharing the same corridor. This section discusses the wind fields on the third floor of D3 under three wind directions.

**Figure 17:**
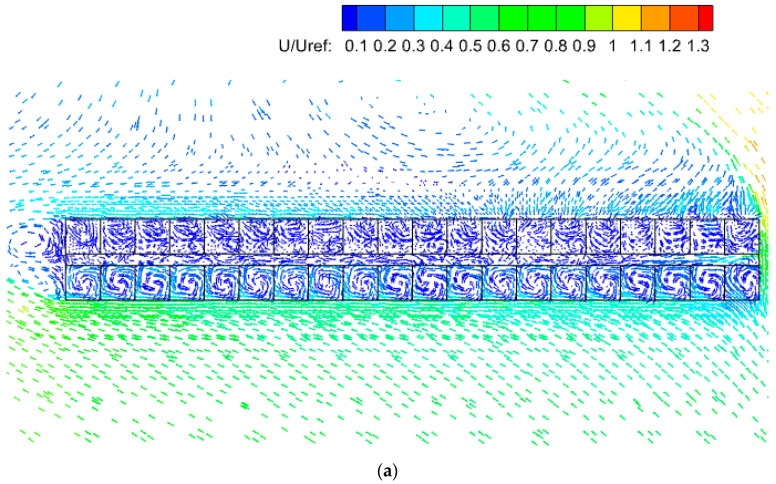

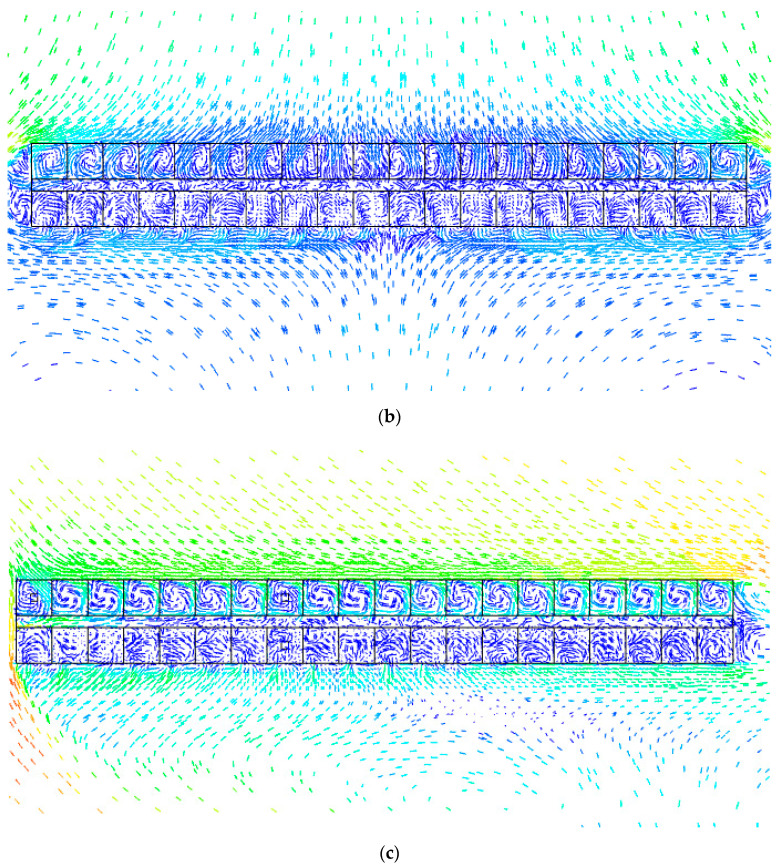
Vectors of mean velocity on horizontal planes around the building under three wind directions at a height of Z = 0.76 m. (**a**) Southeast wind. (**b**) North wind. (**c**) Northwest wind.

As can be seen in Figure 17, with external open windows, a clear vortex occurred in each windward room with relatively larger wind velocities, while in the leeward rooms, the air movement became slower, and vortices were not formed. With internal openings, the rooms on this floor interacted highly through the corridor. The airflow distribution within and around the building was critical to the spread of pollutants between units.

#### 5.2.2. Pollutant Concentrations of Each Room

**Figure 18:**
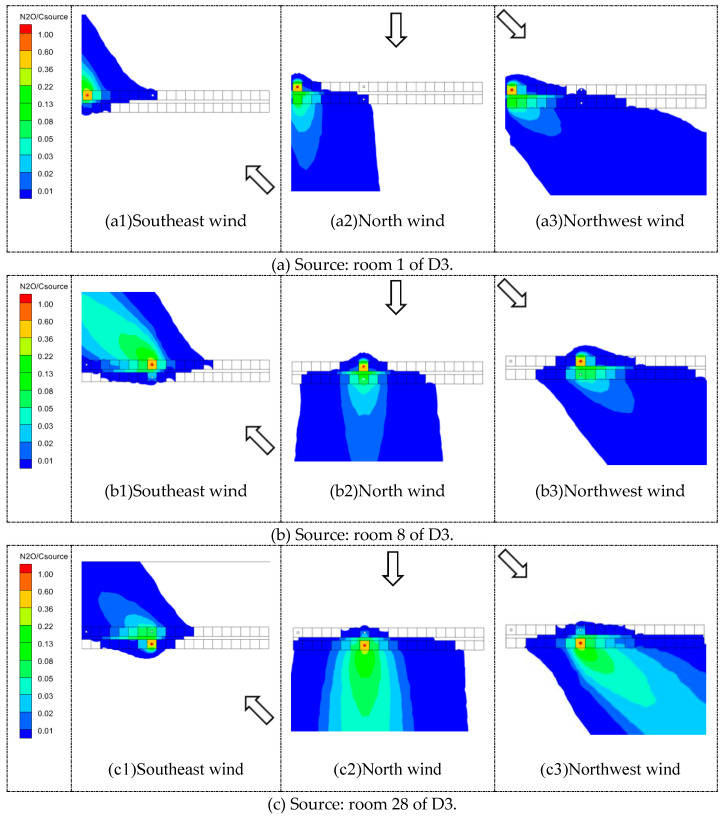
Comparisons of nondimensional concentration fields on horizontal plane Z = 0.76 m with diverse source locations under three wind directions. Arrows represent three different wind directions.

**Figure 19:**
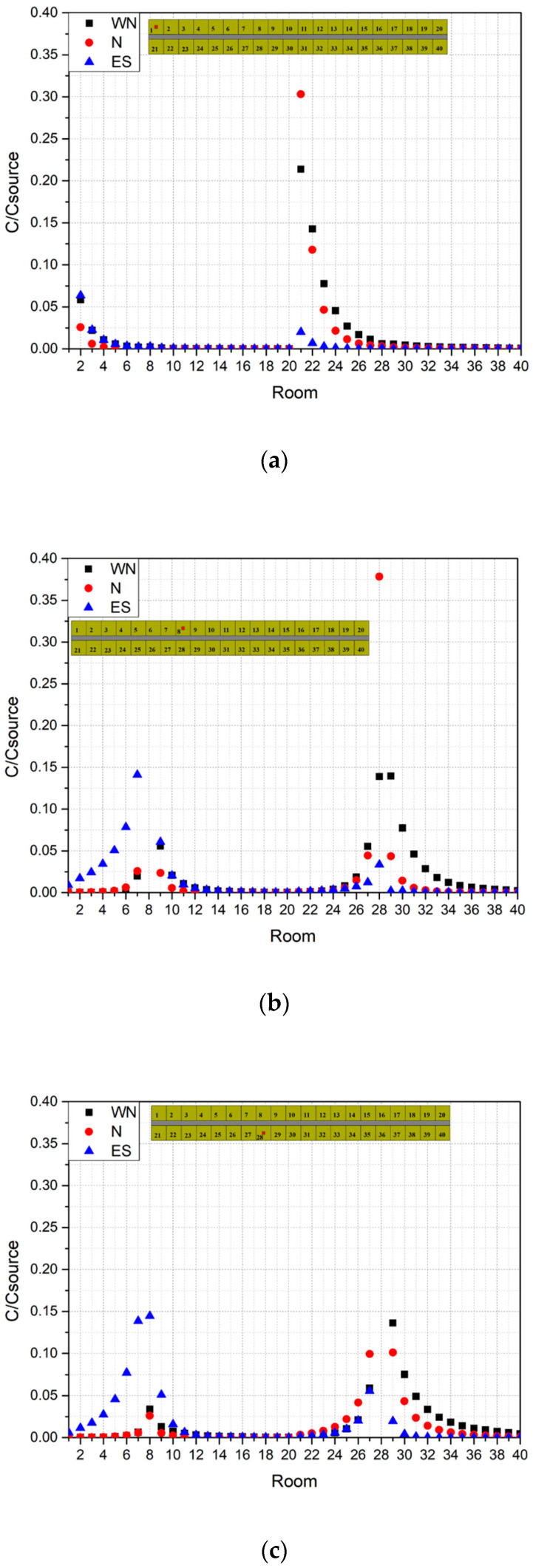
Pollutant concentrations of each room on horizontal plane Z = 0.76 m with three source locations under three wind directions. (**a**) Source 1: Room 1. (**b**) Source 2: Room 8. (**c**) Source 3: Room 28.

Three rooms were chosen as source rooms to investigate the pollutant dispersion inside D3. Figure 18 shows a comparison of nondimensional concentration fields on the horizontal plane with three source locations under three wind directions. Figure 19 shows the nondimensional concentrations of pollutants at the three source locations on the horizontal plane of Z = 0.76 m for the three wind directions. It is clear from Figure 19a that when the pollutant source was room 1 under north and northwest wind directions, the highest pollutant concentration was found in room 21, which was opposite room 1. Similar results can also be found in Figure 19b where, when the pollutant was released from room 8, the largest concentration was found in room 28. In addition, concentrations of the opposite rooms were larger than those in rooms on the same side as the source room. This indicates that the pollutant was most likely to diffuse through the corridor to the room directly opposite when the source was located on the windward side. Figure 18(a2,a3,b2,b3) can further illustrate these findings.

When the source was located on the leeward side, the pollutants released from the source room mainly affected the rooms on the same side, and the concentrations of the opposite rooms were obviously decreased, which can be clearly seen in Figure 19c with the source in room 28 and wind in the north direction (red dot). Similar phenomena can also be found in Figure 19a,b for wind in the southeast direction (blue triangle). In the rooms on the same side, the pollutant concentrations gradually grew lower on both sides of the source room. These findings are consistent with those of Cheng et al. [23]. They further implied that, in a compact dormitory environment, pollutants or viruses can be easily transported among surrounding units, which is highly dangerous, especially during an epidemic outbreak.

## 6. Conclusions

In this study, the airflow characteristics and concentration dispersions of pollutants between and within dormitory complexes under three wind directions were investigated through CFD simulations. N_2_O tracer gas was released from specific units of the building models, and the re-entry ratios of pollutants between the units were analyzed to assess the risk of transmission. The results showed that pollutant transport in and around the buildings was feasible under the three wind directions. Moreover, in the dormitory complex environment, compact space and short distance made the risk of infection higher. Under three wind directions, the airflow patterns around and within the buildings were sensitive to the approaching wind direction. Therefore, the dispersion paths of pollutants could be influenced by the three wind directions. The innovations of this study included two points. Firstly, this study investigated the cross-diffusion pathways of pollutants caused by natural ventilation (both external and internal ventilation windows) in a densely populated building environment (school dormitory) with the CFD method. Then, based on an actual dormitory complex and surrounding buildings, this study considered three realistic wind directions. The conclusions can be drawn as follows:

1. The dispersion of pollutants could occur both vertically and horizontally under three wind directions. The largest re-entry ratios of pollutants were located in the room nearest to the source position along the wind direction.
2. Based on the wind field and ACH results, a change in the three wind directions could affect the upstream building alignment of the target buildings. Changes in the upstream buildings could alter the pattern of airflow around a target building, which resulted in different airflow characteristics and pollutant concentrations. When the wind direction was oblique, the ACH values in most rooms of Q1 and D3 were larger than those for the perpendicular direction.
3. The cross-corridor transmission investigated in the present study implied the outbreak patterns of COVID-19 infection in dormitory buildings. When the source room was located on the windward side, pollutants were most likely to disperse through the corridor and ventilation windows to the room directly opposite. When the source room was located on the leeward side, the pollutants released from the source room mainly affected the rooms on the same side.
4. The Wells–Riley model was used to assess the level of infection risk in the dormitory complex. The results showed that the risk of infection was high when there was little ventilation around the pollutant source room. The biggest risk of infection was when a source room was located on the windward side, and the risk of infection in other rooms on the same side as the source room was large in the windward direction.

## Figures and Tables

**Table 1:** Boundary conditions.

	Power Law Type
Domain inlet	$U=\frac{u^{*}}{\kappa }\ln\left(\frac{z+z_{0}}{z_{0}}\right)$
	$k=\sqrt{C_{1}\cdot \ln\left(z+z_{0}\right)+C_{2}}$
	$\varepsilon =\frac{u^{*}\sqrt{C_{\mu }}}{\kappa \left(z+z_{0}\right)}\sqrt{C_{1}\cdot \ln\left(z+z_{0}\right)+C_{2}}$
Domain outlet	$\frac{\partial }{\partial x}\left(u,v,w,k,\varepsilon \right)=0$
Domain ceiling	$w=0,\frac{\partial }{\partial x}\left(u,v,w,k,\varepsilon \right)=0$
Domain lateral sides	$v=0,\frac{\partial }{\partial x}\left(u,v,w,k,\varepsilon \right)=0$
Domain ground	Standard wall functions
Building surfaces	Non-slip for wall shear stress
Turbulence model coefficients	κ=0.4187 $,\;C_{u}=0.069$

## Data Availability

Not applicable.
